# The effectiveness of a motivational text-messaging program for smoking cessation after coronary angioplasty: a quasi-experimental study

**DOI:** 10.1186/s13104-022-06267-x

**Published:** 2023-01-02

**Authors:** Mohammad Sadegh Mobaraki, Zahra Khademian, Fatemeh Shirazi

**Affiliations:** 1 Student Research Committee, School of Nursing and Midwifery, Shiraz University of Medical Sciences, Shiraz, Iran; 2 Department of Nursing, School of Nursing and Midwifery, Shiraz University of Medical Sciences, Shiraz, Iran; 3 Community Based Psychiatric Care Research Center, Department of Nursing, School of Nursing and Midwifery, Shiraz University of Medical Sciences, Shiraz, Iran

**Keywords:** Angioplasty, Balloon, Coronary, Cardiovascular diseases, Learning, Text messaging, Self-efficacy, Smoking cessation

## Abstract

**Objective:**

Smoking is an important risk factor of coronary artery stenosis after angioplasty. Therefore, this investigation aimed to determine the effectiveness of a motivational text-messaging program for smoking cessation after coronary angioplasty. This quasi-experimental study was conducted on 100 patients after angioplasty. The patients were divided randomly into two intervention and control groups. The intervention group received 32 text messages about smoking cessation for 2 months. The control group received only routine training. The primary and secondary outcomes were success and self-efficacy in quitting smoking cigarettes, respectively. Both groups filled out the related questionnaires before and after the intervention. The data were analyzed using SPSS software version 22 and Chi-square tests, independent t-test, and paired t-test. P < 0.05 was considered significant.

**Results:**

Success in quitting cigarette smoking was significantly higher in the intervention group (n = 29, 61.7%) compared to the control group (n = 2, 4.3%) (p < 0.001). Moreover, after the intervention, the mean score of self-efficacy in the intervention group (11.01 ± 44.75) was significantly higher than the control group (6.51 ± 3.11) and also higher than before the intervention (5.51 ± 2.44) (P˂0.001). The motivational text-messaging program can improve self-efficacy and success in smoking cessation in patients after coronary angioplasty.

**Supplementary Information:**

The online version contains supplementary material available at 10.1186/s13104-022-06267-x.

## Introduction

The high prevalence of coronary artery diseases (CAD) has increased the need to surgical interventions like angioplasty [1]. Despite the expansion and effectiveness of angioplasty, unfortunately, it cannot stop the course of atherosclerosis. So that 20 to 40 percent of patients may experience re-stenosis or angina after angioplasty [2, 3]. Nevertheless, by controlling CAD risk factors, more than 50% of deaths and disabilities caused by these diseases can be reduced [4]. Smoking is one of the most important modifiable cardiovascular risk factors that increases the possibility of recurrence of coronary artery stenosis after angioplasty [5, 6]. The spread of tobacco products is a worldwide problem, particularly in low-income countries [7–9]. In Iran, the prevalence of smoking is reported at least 20.2 percent for men and 0.8 percent for women [10].

Smokers compared to nonsmokers, have a lower level of self-efficacy [11]. Self-efficacy in quitting smoking means the patient’s trust in their ability to quit cigarettes completely [12]. The more people's self-efficacy increases, the more their tendency to quit smoking and the possibility of their success in quitting cigarette augments [13, 14]. Therefore, it is necessary to apply strategies to enrich self-efficacy in smokers.

Nurses have an important role in patient counselling about smoking cessation. However, the limitations of time and resources [15, 16], and the short hospital stay of patents after angioplasty reduces nurses opportunity to participate in these consultations. Therefore, it is necessary to find measures to strengthen the nurses role in this field [17].

Previous studies have investigated the use of text messages (SMS) in improving health behaviors [18] and smoking cessation [19, 20]. Despite the positive views towards smoking cessation SMS, studies have shown problems in the effectiveness and applicability of these messages [19, 20]. Moreover, some people doubted the potential harm of smoking and had a negative attitude towards quitting advices. However, most people prefer messages that motivate them to quit smoking [20, 21].

Individual's attitudes and motivations play an important role in their self-efficacy, treatment adherence, and success in quitting smoking [14, 22, 23, 24]. The majority of smokers consider quitting smoking difficult and feel they are doomed to fail [25]. Allen Carr believes that the reason for people's failure in quitting smoking is that they consider quitting as a difficult and impossible task to achieve. He thought if people had a positive attitude toward quitting smoking the quitting process would be a feasible and enjoyable process for them [22]. However, we did not find sufficient scientific evidence for the effectiveness of an intervention based on his beliefs in the form of SMS. Hence, this study aimed to determine the effectiveness of a motivational text-messaging program for smoking cessation after coronary angioplasty.

## Main text

### Materials and methods

#### Setting and participants

This quasi-experimental study was conducted from July to October 2019 in three cardiac care units in Kowsar hospital, Shiraz, Iran. Using G-Power software and based on a previous study on success in smoking cessation, with P1 = 0.67, P2 = 0.37, α = 0.05, and power = 0.9, a 92-patient sample size was estimated. Therefore, considering the possibility of attrition, 100 patients were selected [26]. The patients were divided into intervention and control groups with a 1:1 allocation (50 people per group) using permutation block randomization. The randomization sequence was generated by an independent researcher in the form of 25 blocks of size 4 using random allocation software. This sequence was put in numbered sealed envelopes. After selecting each eligible participant, the envelope was opened and according to its code, the person was assigned to the intervention or control group. The inclusion criteria included passing eight hours from angioplasty and having stable vital signs, being over 18 years old, having smoking history before being hospitalized, and having a cell phone and the ability to use texting service. The exclusion criteria were addiction to narcotics and consumption of other nicotine products other than cigarettes based on self-report, not filling out the questionnaires completely and the patients’ death.

During the investigation, two people from the intervention group and two people from the control group were excluded from the study because they did not refer to the hospital and did not complete the post-test. Furthermore, one person from the control group was omitted because of death and one person from the intervention group was unwilling to continue the intervention; finally, the data of 94 patients were analyzed.

#### Intervention

In this study, the patients in both intervention and control groups received routine training of the unit from nurses. The routine training included emphasizing the importance of adherence to treatment regimen and controlling modifiable risk factors for CAD.

In the intervention group, a motivational text-messaging program for quitting smoking was applied too. In this way, from the second day after discharge, 32 motivational-educational SMS were sent to the patients. These SMS were designed based on Allen Carr's ideas about an easy way to stop smoking [22]. To ensure the validity of SMS, we revised them according to the comments of 10 experts from community health nursing and medical-surgical nursing. Then they were edited in writing (Additional file 1). To improve patients’ attitudes towards smoking cessation and motivate them in this regards, these messages questioned people's common misconceptions about smoking and the potential risks of quitting. Moreover, according to Allen Carr's ideas, instead of a mere emphasis on threats and dangers of smoking, the benefits of quitting and how deleting cigarettes from life can return valuable assets like energy, joy, peace, health, and financial resources were highlighted [22]. Every week, four messages were sent on certain days from 8 a.m. to 8 p.m. for 2 months. Patients sent the word "received" after receiving each message. In addition, every week, the researcher sent a message to check whether the patients were following the SMS.

#### Data collection

Data collection tools were Persian versions of the smoking status questionnaire, success in quitting smoking and Smoking Abstinence Self-Efficacy Questionnaire (SASEQ). The pre-test was done when the hospitalized patients’ condition was stable after angioplasty. After the intervention was finished, the post-test questionnaires were completed when the patients referred to the outpatient clinic for their scheduled appointment.

The smoking status questionnaire was extracted from the smoking questionnaire related to the Multinational Monitoring of Trends and Determinants in Cardiovascular Disease (MONICA) project. This questionnaire has 13 questions to examine the smoking condition [22, 27]. Kazemzadeh et al. selected eight questions about active smoking from this tool and after translation, investigated its psychometric properties. Similarly, in the present study, these questions were used [26].

To investigate the self-efficacy of people for quitting smoking the SASEQ designed by Spek et al. was used. The questionnaire has six questions and the answers are based on 5-point Likert scale scored from "I surely won't smoke" [28] to "I'd surely smoke" (0). The obtained score would be a number between 0 to 24. A higher score shows higher self-efficacy in quitting smoking [29]. Spek et al. in addition to factor analysis, signified the discriminant validity of the tool via its negative and weak relationship with the Edinburgh Depression Scale (*r* =  − 0.145, *p* = 0.001). Furthermore, they confirmed its reliability by internal consistency (Cronbach’s alpha = 0.89) [29].

To investigate success in quitting cigarette smoking, the self –report 4-weeks quitting (SR4WQ) questionnaire was used [26, 30]. In this questionnaire, people were first asked about cigarette smoking during the past two weeks and if their answers were yes, four questions were asked about the reasons of return to smoking. People who stated that they have completely quit cigarette smoking were considered successful. Quitting smoking by 50% of the people indicates the success of the intervention.

Kazemzadeh et al. confirmed the content validity of Persian versions of three tools by offering them to 10 experts. Additionally, they confirmed the reliability of the smoking status questionnaire by test re-test (r = 0.86), the reliability of SR4WQ by Cronbach’s Alpha of 0.87 [26], and SASEQ reliability by Cronbach’s alpha of 0.94 [31].

#### Data analysis

Data were analyzed using SPSS software version 22. The research variables were described using descriptive statistics. To compare the two groups in terms of qualitative variables, the Chi-square test was used. Also, to compare the two groups regarding quantitative variables, the independent t-test was applied. For comparing inter-group self-efficacy, a paired t-test was employed. P < 0.05 was considered as the statistical significance level for all analyzes.

## Results

The participants were 94 patients with mean age of 58.9 ± 9.23 (57.9 ± 9.25 years in the intervention group and 59.89 ± 8.65 years in the control group, p = 0.1). Most of the participants were male and married. There was no significant difference between the intervention and control groups in terms of demographic variables and smoking status before the intervention. (Tables 1 and 2).

**Table 1:** Baseline characteristics of the two intervention and control groups

Group		Total	Intervention	Control	P-value
Variable		Frequency (%)	Frequency (%)	Frequency (%)	
Sex	Male	93(98.9)	47(100)	46(97.9)	0.5*
	Female	1(1.1)	0(0)	1(2.1)	
Education	Academic	16(17)	6(12.8)	10(21.3)	
	Secondary	30(31.9)	16(34)	14(29.7)	0.471*
	Elementary and secondary school	48(51.1)	25(53.2)	23(49.1)	
Marital status	Single	3(3.2)	1(2.1)	2(4.3)	0.557*
	Married	91(96.8)	46(97.9)	45(95.7)	
Occupation	Self-employed, housewife, worker	53(56.4)	25(53.2)	28(59.5)	0.914*
	Employee and retired	41(43.6)	22(46.8)	19(40.4)	
History of myocardial infarction	Yes	13(14.1)	6 (13)	7 (15.2)	0.765*
	No	79(85.9)	40 (87)	39(84.8)	

^*^Chi-square test

**Table 2:** Between-group comparison of the smoking status before the intervention

Group		Intervention	Control		Group	Intervention	Control	
Variable		Number (percentage)	Number (percentage)	P-value*	Variable	Mean ± SD	Mean ± SD	P-value**
Have smokers in the family	Have	3(6.4%)	12(25.5%)	0.11	Number of cigarettes smoked daily	18.64 ± 10.63	17.64 ± 9.89	0.638
	Do not have	44(93.6%)	35(74.5%)					
Quitting cigarette contest	Have	43(91.5%)	46(97.9%)	0.361	The age of starting smoking cigarettes	19.96 ± 4.4	19.87 ± 3.8	0.92
	Do not have	4 (8.5%)	1(2.1%)					
Number of smoking days in a week	2–4 days	5(10.6%)	4(8.7%)	0.99	Number of quitting attempts	1.94 ± .99	1.81 ± .71	0.474
	Everyday	42(89.4%)	42(91.3%)					
Continuing to smoke cigarettes	Always	40(85.1%)	41(87.2%)	0.99	The most number of cigarettes smoked in a year	22.87 ± 22.61	22.34 ± 22.34	0.818
	Sometimes	7(14.9%)	6(12.8%)					

^*^Chi-square test

^**^Independent T-test

Besides, the pretest scores of the smoking abstinence self-efficacy were not significantly different between the intervention (5.51 ± 2.44) and control groups (5.02 ± 1.37) (p = 0.23). However, the post-test score of the intervention group (11.01 ± 4.75) was significantly higher than the control group (6.51 ± 2.95). Furthermore, the post-test scores of the intervention and control groups were higher than their pre-test scores (p ≤ 0.001).

Moreover, after the intervention, 61.7% of individuals from the intervention group and 4.3% of people from the control group succeeded to quit smoking and the between-group difference in success in smoking cessation was statistically significant (P < 0.001) (Table 3).

**Table 3:** The comparison of success in smoking cessation between the intervention and control groups

Group		Intervention	Control	P-value*
Variable		Number (%)	Number (%)	
Success in smoking cessation	Yes	29(61.7)	2(4.3)	< 0.001
	No	18(38.3)	45(95.7)	
Smoking cigarettes because of anger	Yes	18(100)	27(60)	< 0.001
	No	0(0)	18(40)	
Smoking due to being exposed to stimulating situations	Yes	6(33.3)	24(53.3)	0.151
	No	12(66.7)	21(46.7)	
Smoking due to stress	Yes	17(94.4)	37(82.2)	0.268
	No	1(5.6)	8(17.8)	

^*^Chi-square

## Discussion

The findings showed that a motivational text-messaging program resulted in the improvement in smoking cessation and self-efficacy in patients after coronary angioplasty.

The positive effect of consultative-educational interventions like educational SMS was reported in previous studies as well [32, 33]. Similarly, a study conducted in South Korea revealed the effectiveness of telephone consultations and SMS for patients with CAD in quitting smoking and increasing their self-efficacy [32]. Another Iranian study also showed the positive effect of an educational-consultative program on the success and self-efficacy in quitting smoking in patients with acute coronary syndrome [26]. In accordance with our findings, interventions like education and following-up based on Allen Carr’s method boosted the rate of quitting cigarette smoking in previous studies [34–36].

Although it is expected that patients refuse to start smoking again after angioplasty due to the fear of disease recurrence, the results of this study showed that only 4.3% of the patients in the control group had quit smoking 2 months after angioplasty. This shows that without purposeful programs, the rate of smoking abstinence might be low, and it is necessary to apply a deliberately designed intervention to prevent the individuals’ return to smoking.

Patients considered anger and stress to be the most important factors for continuing smoking. In this regard, a systematic-review showed positive effects of psychological interventions on quitting smoking in patients with CAD [37]. Therefore, to attain more favorable results, it is required to apply interventions to improve stress and anger management, in addition to motivational-educational SMS.

Among the strong points of this study, is the use of two-groups before and after design that made the comparison of intra-group and inter-group changes possible. Utilizing the block randomization increased the validity of the study as well. The use of SMS intervention based on Allen Carrr's beliefs was another strength of the study.

## Conclusion

The current findings revealed that a motivational text-messaging program could enhance patients’ self-efficacy and success in quitting smoking after coronary angioplasty. Hence, we recommend that similar programs be performed to help these patients in quitting smoking. Moreover, examining the effectiveness of combined programs containing motivational SMS and psychological interventions is highly recommended.

## Limitation

Data were collected by self-report, so there is the possibility of desirability bias. Besides, because of time limitations related to the thesis proposal, we investigated the short-term outcomes of the intervention; a longer follow-up period would reveal its long-term effects. Moreover, due to the nature of the intervention, the patients were aware of their assigned groups and so blinding was impossible. Finally, the study consisted of mainly male participants. Hence, the findings are not generalizable to females.

## Supplementary Information

**Additional file 1: **The text messages used in the present investigation.

## Data Availability

Data are available from corresponding author upon request.

## Abbreviations

CAD: Coronary Artery Diseases
MONICA: Multinational Monitoring of Trends and Determinants in Cardiovascular Disease
SASEQ: Smoking Abstinence Self-Efficacy Questionnaire
SMS: Text Messages
SPSS: Statistical Package for the Social Sciences
SR4WQ: Self–Report 4-Weeks Quitting
