# Supplementary material for: The effectiveness of a motivational text-messaging program for smoking cessation after coronary angioplasty: a quasi-experimental study
Source: BMC Res Notes. 2023 Jan 2;16:1. doi: 10.1186/s13104-022-06267-x (PMC9809064; doi:10.1186/s13104-022-06267-x)
Supplement: Supplementary file 1 — Additional file 1: The text messages used in the present investigation. [file 13104_2022_6267_MOESM1_ESM.docx]

**Supplementary file 1. The text messages used in the present investigation**

| 17. Did you know that if you stop smoking for just three days, 100 percent of the nicotine entered into your body would be cleaned? | 1. Do you believe that nonsmokers live a better life than smokers do? |
| --- | --- |
| 18. Although nicotine is a highly addicting poison, as soon as you put out your last cigarette, there would only be 3 percent of that in your body after 8 hours. | 2. What is your answer to this question: How do nonsmokers live comfortably and independently without smoking cigarettes? |
| 19. You only need three weeks to get rid of the physical and mental effects of cigarette smoking. | 3. If we make a comparison between nonsmokers and smokers in an equal stressful situation, nonsmokers will be less anxious. |
| 20. Why did you start smoking cigarettes? Because of friends? Problems? Now that you realize that you were a victim, why don’t you quit it? | 4. Cigarettes relieve tiredness, if this belief is true, then smokers should never get tired. |
| 21. If you want to quit smoking cigarettes, put away all matchsticks, lighters, and ashtrays. | 5. Some people believe that cigarettes are calming and remove tension and anxiety, while cigarette smoking increases stress and anxiety. |
| 22. As soon as you decide to smoke your last cigarette, you change to a nonsmoker. | 6. If you claim that smoking a cigarette refreshes you and relieves your tiredness, then why do you smoke it when you are not tired? |
| 23. Right now that you have made your decision to quit smoking, your success is guaranteed. | 7. Increased concentration and energy caused by smoking a cigarette is nothing but a big lie, if it was true, smokers would be more energetic and fresher people with better brain performance than nonsmokers would. |
| 24. Quitting cigarette smoking is not a difficult task, millions of people have done it successfully, and so can you. | 8. Estimate the expenses of cigarette smoking in one year! You see that by smoking you burn away a good amount of financial investment and endanger your health too. The expenses of curing diseases caused by smoking are high. |
| 25. The only person who makes you light the next cigarette is just you, yourself. | 9. Perhaps smoking cigarettes has become such a routine trivial matter for you that you do not realize how much of your valuable assets like money, health, happiness, energy, and peace of mind have been lost. |
| 26. The first step to quitting smoking is not lighting up the next cigarette. | 10. Did you know that pale lips, wrinkled and dull skin of the face that you see in smokers are all due to artery occlusion in their body? |
| 27. Don’t tell yourself, I should not smoke cigarettes anymore, instead say: is not it great that I do not smoke and I am no longer a slave to nicotine! | 11. Smoking cigarettes is more a habit and addiction than an act of joy; remember the first pack of cigarettes, how disgusting it was! |
| 28. If you examine positive and negative aspects of smoking cigarettes, you would always come to one result, and that is: do not smoke and be happy forever. | 12. Sometimes because you are trapped in smoking, to tolerate this new condition, you have to close your eyes on all its negative effects. |
| 29. Your pains and suffering after quitting are signs of your recovery, you’ll reach peace of mind much sooner than you imagine. | 13. If you do not see any words saying tranquility, concentration, refreshing, …printed on cigarette packs, that’s because they are not true. |
| 30. Never use substitutions for cigarettes, who are looking for a substitute for the biggest cause of death in the world? | 14. You have two choices: smoke and base your life on disease and addiction, or quit smoking cigarettes and take control of your life and replace your disease and addiction with happiness and health. |
| 31. Smoking cigarettes is a disease, you don’t need to find a replacement like chewing gum, nicotine sticks, or spray for it when you get rid of your disease. | 15. To quit cigarette smoking, forget about doubts, all researches done so far show that cigarettes ruin the body. |
| 32. Enjoy every nonsmoking moment and celebrate freedom from nicotine. | 16. If you smoke a cigarette occasionally then keep in mind that you will become more addicted than smokers, because you will become involved with the negative side effects and also, lose the motivation to quit it. |
